# Plants Developed by New Genetic Modification Techniques—Comparison of Existing Regulatory Frameworks in the EU and Non-EU Countries

**DOI:** 10.3389/fbioe.2019.00026

**Published:** 2019-02-19

**Authors:** Michael F. Eckerstorfer, Margret Engelhard, Andreas Heissenberger, Samson Simon, Hanka Teichmann

**Affiliations:** ^1^Department Landuse and Biosafety, Environment Agency Austria, Vienna, Austria; ^2^Federal Agency for Nature Conservation, Bonn, Germany

**Keywords:** new genetic modification techniques, nGM, genome editing, regulation, biosafety, risk assessment, regulatory trigger

## Abstract

The development of new genetic modification techniques (nGMs), also referred to as “new (breeding) techniques” in other sources, has raised worldwide discussions regarding their regulation. Different existing regulatory frameworks for genetically modified organisms (GMO) cover nGMs to varying degrees. Coverage of nGMs depends mostly on the regulatory trigger. In general two different trigger systems can be distinguished, taking into account either the process applied during development or the characteristics of the resulting product. A key question is whether regulatory frameworks either based on process- or product-oriented triggers are more advantageous for the regulation of nGM applications. We analyzed regulatory frameworks for GMO from different countries covering both trigger systems with a focus on their applicability to plants developed by various nGMs. The study is based on a literature analysis and qualitative interviews with regulatory experts and risk assessors of GMO in the respective countries. The applied principles of risk assessment are very similar in all investigated countries independent of the applied trigger for regulation. Even though the regulatory trigger is either process- or product-oriented, both triggers systems show features of the respective other in practice. In addition our analysis shows that both trigger systems have a number of generic advantages and disadvantages, but neither system can be regarded as superior at a general level. More decisive for the regulation of organisms or products, especially nGM applications, are the variable criteria and exceptions used to implement the triggers in the different regulatory frameworks. There are discussions and consultations in some countries about whether changes in legislation are necessary to establish a desired level of regulation of nGMs. We identified five strategies for countries that desire to regulate nGM applications for biosafety–ranging from applying existing biosafety frameworks without further amendments to establishing new stand-alone legislation. Due to varying degrees of nGM regulation, international harmonization will supposedly not be achieved in the near future. In the context of international trade, transparency of the regulatory status of individual nGM products is a crucial issue. We therefore propose to introduce an international public registry listing all biotechnology products commercially used in agriculture.

## Introduction

Genetically modified (GM) crop plants developed by recombinant DNA (rDNA) technology are regulated in most countries by biosafety frameworks established by specific legislation. These biosafety frameworks typically build on the fundamental principles for food and feed safety and the environmental risk assessment of crops produced by modern biotechnology developed e.g., by international bodies like the FAO/WHO and the OECD (Jones, 2015a). Particularly important for the development and international harmonization of biosafety frameworks is the Cartagena Protocol on Biosafety (CPB), established under the Convention on Biological Diversity. The Parties to the CPB, currently 171 countries, have the obligation to follow the provisions laid down in the Protocol, when developing their biosafety regulations.

In parallel to classic GM technology a wide range of “new genetic modification techniques” (nGMs) was developed for the (genetic) modification of organisms, including plants, for research purposes or for the development of crops for agricultural use. These nGMs are also referred to as “new techniques” or “new breeding techniques” in other sources (Lusser et al., 2012; Vogel, 2016; SAM, 2017). For the purpose of clarification and to avoid the possible misconception on the part of non-experts that these technologies are just variants of conventional cross-breeding methods we do not use these terms in this paper.

The range of nGMs addressed in this paper includes the following techniques:

- Genome editing with site-directed nucleases (SDNs), e.g., using clustered regularly interspaced short palindromic repeat (CRISPR)-directed nucleases, transcription activator-like effector nuclease (TALENs), zinc-finger nucleases (ZFNs) or meganucleases. SDN-based techniques can also be applied for multiplex genome editing and “base editing” as well as for modification of transcriptional regulation.
- Genome editing directed by synthetic oligonucleotides, also referred to as oligonucleotide-directed mutagenesis (ODM)
- RNA directed DNA methylation, an approach for modifying epigenetic regulation of gene expression
- Cisgenesis and intragenesis
- Transgrafting, in particular the use of GM-rootstocks in grafting
- Agro-infiltration
- Haploid induction and accelerated breeding, i.e., examples of techniques developed to assist complex breeding schemes

Currently particular focus is placed on nGM applications for genome editing, which involve the use of SDNs, like CRISPR/Cas9 (Wolt et al., 2016a). These genome editing approaches are deemed relevant for the future development of crop plants due to their practical advantages, like the wide range of potential applications concerning different plant species and traits (Voytas and Gao, 2014; Bortesi and Fischer, 2015). Genome editing by SDNs may be used in different ways and to achieve different objectives (Schiml and Puchta, 2016). Generally there are three types of SDN-approaches: (i) applications to introduce random changes to the genomic DNA sequence at specific locations, which are created by error-prone repair of double-strand breaks introduced by a particular SDN (SDN-1 type applications); (ii) applications based on homology-dependent repair of site-specific double-strand breaks to introduce small specific sequence changes at genomic targets instructed by DNA-template sequences which are supplied *in trans* (SDN-2 type applications); (iii) applications based on homology-dependent introduction of larger-sized DNA elements of heterologous origin into the recipient genome at specific locations (SDN-3 type applications).

Besides these basic types of genome editing a number of additional approaches were developed recently. At the one hand CRISPR-based systems directed by multiple guide RNAs can be used for simultaneous modification of different genomic target loci (“multiplex genome editing”). On the other hand modified SDNs with a disabled nuclease function can be employed to introducing specific sequence changes via directed chemical modification of nucleobases in an intact strand of DNA (“base editing”) and to modifying the transcriptional regulation of gene expression (“epigenome editing”) (Puchta, 2016; Tycko et al., 2017; Rees and Liu, 2018).

Currently a lively discussion is underway in many countries concerning the regulatory approach toward crops generated by nGMs and in particular for applications of genome editing (Jones, 2015b; Huang et al., 2016; Wolt et al., 2016b). The debate is fuelled on the one hand by a significant interest of the research and development community in these technologies and their wide range of application in plant development. Furthermore, widespread public interest is focused on nGMs and genome editing because the application of these biotechniques in plant development is challenging existing regulatory paradigms for plants produced by biotechnology (Wolt and Wolf, 2018). Discussed in these respect are similarities and differences of nGMs from either classic GM-technology or conventional breeding approaches. Such debates are conducted at both the national and international levels, including the CPB or the OECD (OECD, 2016, 2018), and involve a wide range of stakeholders, including regulators, scientists, industry and non-governmental organizations.

The main question addressed in these discussions is whether products generated by different nGMs should be subject to existing biosafety frameworks. A closely related issue relevant for all regulatory frameworks is which risk assessment and risk management approaches are deemed appropriate for nGM applications (Wolt, 2017). This question is relevant for all countries.

The ongoing general discussion also addresses other related issues: Which monitoring, labeling and traceability requirements should nGM products be subject to? How can coexistence be ensured between biotechnology and non-biotechnology plants? Should a broader assessment of sustainability and socio-economic issues, e.g., as conducted in some countries for GMOs, be implemented for nGM applications? Such questions are highly relevant for regulatory frameworks, which implement these requirements for GMOs, among them the EU. Because these issues are not directly connected with premarket risk assessment and not all countries implement such requirements, we do not focus on these questions in this publication. We, however, want to underline that these issues merit in-depth consideration in their own right and need to be further addressed.

In relation to risk assessment regulatory challenges associated with the application of nGMs arise as a result of their specific methodological characteristics and the broad range of different products which may be developed using these techniques:

- nGMs may be used to generate different types of genetic and epigenetic modifications. Different qualities and quantities of modifications need to be taken into account, ranging from random or directed changes of the DNA sequence at specific genomic locations (SDN-1 and SDN-2, respectively), to insertion of larger-sized DNA elements of heterologous origin into the recipient genome at specific or random locations (SDN-3 and cisgenesis/intragenesis respectively), and transient or heritable changes of epigenetic modifications (epigenome editing).
- nGMs are associated with a different potential to introduce unintended modifications which are either linked to characteristics of the particular nGM method or to other biotechnological techniques used throughout the development process (e.g., methods for *in vitro* plant cell cultivation, the generation and use of plant protoplasts and the regeneration of viable plants from single cells or tissue cultures) (Zheng and Gu, 2015; Bortesi et al., 2016). Depending on the phenotypical outcomes of such unintended modifications, they may lead to adverse phenotypical effects.
- nGMs may be used to create a variety of different traits. Those traits might already be present in natural populations and/or agronomically used plant varieties or may be novel in terms of agricultural use (HCB, 2017).

Thus, different sources of potential hazards need to be considered to assess whether applications developed by specific nGM approaches are associated with relevant risks (Eckerstorfer et al., 2014). Such hazards may be associated directly with specific new trait(s) e.g., herbicide resistance traits which are associated with adverse environmental impacts (Schütte et al., 2017). Hazards can also be indirectly associated with the intended modifications if these changes have additional unintended phenotypes. An example are crops developed by genome editing with increased disease resistance due to the knockout of certain (*mlo*) disease susceptibility genes, which are also involved in other physiological functions in addition to their role in fungal pathogenicity (Kusch and Panstruga, 2017). Another source of potential hazards are unintended changes introduced throughout the process of developing a final product by a particular nGM or a combination of biotechnological methods, e.g., nGMs developed by genome editing may be associated with adverse effects if off-target modifications at genomic sequences other than the targeted loci result in significantly negative phenotypical changes (Zhao and Wolt, 2017). The possibilities that hazards may be associated with nGM applications and particularly with genome editing applications are discussed in more detail by Eckerstorfer et al. (2017). Therefore, the regulatory frameworks in different countries need to provide appropriate and workable procedures for regulation and risk assessment to address a diverse range of risk issues which may be associated with certain nGM applications. The question which regulatory trigger is implemented in a particular biosafety framework is a matter of crucial importance in this context.

Existing biosafety frameworks for the regulation of GMO use different regulatory triggers, i.e., definitions specifying the products covered by the regulatory frameworks. Such regulatory triggers either refer to specific characteristics of regulated products and the newly developed traits (product-oriented regulatory triggers) or the use of certain technologies in the generation of regulated products (process-oriented regulatory triggers). What both regulatory regimes have in common is that the risk associated with the regulated product, i.e., the modified organism, needs to be evaluated. A key question is if process- or product-oriented regulatory triggers might be better suited for the regulation of biotechnological products in general and of nGM applications in particular (Voytas and Gao, 2014; Kuzma, 2016b; McHughen, 2016; Sprink et al., 2016a). To address this question we analyzed some features of the different regulatory frameworks currently implemented in European and non-European countries with the aim to inform the further discussion on the subject in the EU.

## Analysis and Comparison of Different European and Non-European Biosafety Frameworks

Our study investigates the differences and similarities of regulatory frameworks for biosafety in Argentina, Australia, Brazil, Canada, the EU, New Zealand, Norway, South Africa, Switzerland, and the USA. In particular we examine how nGM applications are covered and regulated by these frameworks, including the general requirements for risk assessment. Furthermore, we analyse current reviews of these systems and proposed amendments, in particular those which are developed to better address nGM applications.

Our comparison of the different frameworks is based on available literature analyzing and explaining the existing legislation related to regulation of biotechnology products in general and nGM applications in particular. To update and complement this information we conducted interviews with regulators and/or experts involved in risk assessment according to existing biosafety legislation. The interview partners answered our questions in a personal capacity based on the understanding that no transcripts of the interviews would be published and that no direct quotes from the interviews would be attributed to specific persons. The information from the interviews provided a background against which previously published information was checked for correctness and validity (September 2017) (Supplementary Material).

Contrary to previous analyses (Schuttelaar, 2015; NAS, 2016; Sprink et al., 2016a; Academy of Science of South Africa, 2017) we did not specifically focus on the regulatory status of emerging nGM applications (i.e., whether specific nGM applications are subject to a particular biosafety legislation framework or not), but on the experience with existing regulatory approaches and their procedures for risk assessment as well as on possible implications for nGM applications.

The studied biosafety frameworks are embedded in different legislative environments and all of the respective countries have actively been implementing these regulations for many years. The USA, Canada, Argentina, Brazil, South Africa, and Australia are among the main producers and exporters of agricultural GM products (ISAAA, 2016). In all of the selected countries an active discussion on how to deal with future regulation of nGM applications is underway at the national level.

Most of the surveyed regulatory biosafety frameworks were introduced in the 1980s and 1990s with the aim to regulate biotech products, in particular products generated by GM-technology (see overview in Table 1).

**Table 1:** Regulatory frameworks for biotechnology analyzed in this study—Legal foundations, characteristics and regulatory requirements for unconfined release e.g., for commercial cultivation or the marketing of regulated biotechnology products.

**Country**	**Main biosafety legislation**	**Framework or specific law (for env. release)**	Regulatory trigger^*^	**Regulatory requirements for unconfined environmental release**	**Authorisation period (for marketing)**
European Union	Biosafety Directives and Regulations (food/feed, env. release) (1990, updated 2001/2003)	Dir 2001/18/EC, supplemented by implementing regulations and GM food and feed regulation (2003)	Process-oriented^**^	Risk assessment, risk management, coexistence, monitoring, labeling, detection methods	10 years, renewable
Argentina	Regulation Framework for Agricultural Biotechnology (1991)	Supplementary Resolution for release of GMOs	Process-oriented	Risk assessment, socio economic considerations	Not limited (possibility of revocation)
Australia	Gene Technology Act (2000), Food Standards Australia New Zealand Act (1991)	Supplementary Regulations e.g., Gene Technology Regulation (2001)	Process-oriented	Risk assessment, risk management and monitoring	Not limited (possibility of revocation)
Brazil	Biosafety law (1995; updated 2005)	Biosafety Law supplemented by implementing Resolutions	Process-oriented	Risk assessment, coexistence, monitoring, labeling, optional socio economic considerations	Not limited (possibility of revocation)
Canada	Regulatory Framework for Biotechnology (1993)	Framework includes regulations for plants with novel traits and novel foods and feeds	Product-oriented (novelty- and risk-based)	Risk assessment, stewardship (risk management)	Not limited (possibility of revocation)
Norway	Gene Technology Act (1993)	Regulations for risk assessment	Process-oriented	Risk assessment, risk management, monitoring, labeling, detection methods, socio-economic/sustainability assessment	10 years, renewable
New Zealand	Hazardous Substances and New Organisms Act (1996), Food Standards Australia New Zealand Act (1991)	Supplementary Regulations (1998, 2003) and Methodology Order (1998)	Process-oriented	Risk assessment, risk management, monitoring (for conditional releases)	Not limited (possibility of revocation)
South Africa	GMO Act (1997)	GMO Regulations (amended in 2010)	Process-oriented	Risk assessment, monitoring, labeling, detection methods, optional socio economic considerations	Not limited (possibility of revocation)
Switzerland	Gene Technology Act (2003)	Release Ordinance (2008)	Process-oriented	Risk assessment, monitoring, labeling, detection methods	10 years, renewable
USA	Coordinated framework for the regulation of biotechnology (1986)	Framework refers to relevant sectoral legislation (e.g., Plant Protection Act, Federal Insecticide, Fungicide, and Rodenticide Act, Toxic Substances Control Act)	Product-oriented (risk-based)	Risk assessment	Not limited (possibility of revocation)

* Classification of the regulatory triggers is not based on legal determination, but according to an assessment by the authors, based on information in the literature and information gathered from interviews with regulatory experts.

** *Classification is disputed, some sources claim that the trigger is both process- and product-oriented (BVL, 2015; Kahrmann et al., 2017)*.

The majority of the countries (Argentina, Australia, Brazil, New Zealand, Norway, and South Africa, Switzerland) and the EU established new sectoral legislation for applications of biotechnology, along with specific regulations for implementation. The biosafety laws or Gene Technology Acts were informed by early work at the international level, e.g., work undertaken by the OECD (OECD, 1986) or work on drafting the CPB. The biosafety framework adopted by the EU in 1990 was also influential in the subsequent adoption of biosafety laws in other European and non-European countries, e.g., South Africa.

The respective laws define the scope of the regulations and provide definitions of products or organisms, which, in the opinion of regulators, stakeholders, and literature, are considered to be mostly process-oriented. The EU definition is widely considered to be process-oriented especially by a number of legal experts (Krämer, 2015; Spranger, 2015). Other experts are of the opinion that the EU-trigger is both process- and product-oriented (Kahrmann et al., 2017).

On an international level the CPB includes a definition for “living modified organisms,” which is also widely considered to be a process-oriented regulatory trigger. The CPB trigger is based on a slightly different wording compared to the definitions used in the EU and other mentioned countries. The USA, Australia, Argentina and Canada are not Parties to the Protocol. Therefore, full compatibility with the CPB is not an issue for these countries. However, the trigger definition for the biosafety law in Argentina is very similar to the trigger of the CPB. All other countries included in this study and the EU are Parties to the CPB and their legislation has either been compatible with the CPB since it entered into force in 2003, or respective changes have been introduced later to establish compatibility, e.g., 2005 in Brazil.

The USA and Canada have used (and updated) existing national legislation to establish regulatory frameworks for biotechnology applications. These countries use product-oriented triggers to define regulated products; however, different triggers have been adopted in the USA and in Canada.

### General Similarities of and Differences Between the Biosafety Frameworks

The analyzed biosafety frameworks were established against different national legislative backgrounds. One of the main differences was the decision on whether to use and adapt existing legislation for biosafety regulation, as in the USA and in Canada, or to establish new sectoral biosafety legislation. On a global level most countries, including the other analyzed countries and the EU, have taken the latter approach.

Both approaches have specific consequences, particularly for developers and regulators. When using existing legislation and established regulatory authorities, the administrative system for regulating biotech products is typically better aligned with existing procedures and statutory responsibilities for non-biotech products. This can result in higher consistency concerning the risk assessment of biotech and non-biotech products as e.g., in Canada. On the other hand the developers need to deal with a number of different statutory authorities which are responsible for different regulatory issues, e.g., the environmental release of modified plants and animals or placing on the market of biotech foods and feeds, e.g., as in the USA and Canada. In both countries several different authorities are involved in the regulation of products.

In the USA the “Coordinated Framework for the Regulation of Biotechnology” has been established 1986 by the White House Office of Science and Technology Policy and updated in 1992 and in 2017 (NAS, 2016; EOP, 2017). It was introduced with the aim of coordinating the regulatory responsibilities of several federal agencies under their existing statutes (Wolt and Wolf, 2018): USDA-APHIS is responsible for applications related to the import, interstate movement, as well as environmental release for field trials and unrestricted cultivation, US-EPA is responsible for products with plant-incorporated protectants and GM microbial pesticides and FDA covers food safety issues and the safety of biotechnological products for medical use.

In Canada the Canadian Food Inspection Agency (CFIA) is responsible for the environmental release of ‘Plants with Novel Traits‘ (PNTs), including PNTs developed with biotechnology methods, and the use of feedstuffs derived from PNTs. Environment Canada is responsible for GM microorganisms and Health Canada for the safety of novel (biotech) foods, respectively (Shearer, 2014; Smyth, 2017).

It was noted that split responsibilities may create complex regulatory pathways and may result in difficulties for product developers to navigate the system (Kuzma, 2016a). For example in the USA US-FDA is providing regulatory oversight for a range of modified animals (as “New Animal Drugs”), that are developed for purposes comprising environmental release. This includes e.g., GM salmon (AquAdvantage), which was authorized for land-based production in 2018, GM mosquito products intended to reduce the vectoring capacity for viruses or other pathogens of these insects or their pathogen load and organisms which qualify as “animals with intentionally altered genomic DNA” under FDA's “Veterinary Innovation Program” (FDA, 2018). In contrast GM mosquito products modified for population suppression are regulated by US-EPA since October 2017^1^. To ensure consistent regulation and to avoid duplication of efforts, close coordination between the involved authorities is necessary. Procedures to address these issues have been implemented in Canada and the USA (EOP, 1992, 2017; Shearer, 2014; NAS, 2016), they include e.g., the possibility for developers to engage in pre-submission consultations with the authorities to address questions regarding the regulatory status of specific products, as well as regulatory and information requirements for risk assessment (Shearer, 2014).

Introduction of new sectoral legislation for biosafety regulation is typically coupled with the establishment of specific lead authorities with consolidated responsibility for all types of biotech products. According to the opinion of some interviewed regulatory experts this might be less confusing for applicants as far as the specific biosafety requirements are concerned, e.g., regarding risk assessment. However, this does not necessarily grant an easier route to quicker decision making on applications submitted for authorization, the EU regulatory framework being an example in point. Decision making in the EU is based on a highly complex and time-consuming procedure involving the European Commission and all Member States, once the risk assessment has been conducted under the lead of the European Food Safety Authority (EFSA) (Hartung and Schiemann, 2014).

### All Investigated Regulatory Frameworks Are Risk-Oriented

All analyzed biosafety frameworks establish a risk-oriented regulatory approach. A mandatory risk assessment is conducted for all regulated products prior to authorization for environmental release or food and feed use with the general intention to ensure environmental and health safety (McHughen, 2016).

Consequently the regulatory triggers of the different regulatory frameworks relate to risks in direct or indirect ways:

- The triggers used in the USA are directly related to specific risk issues: A modified organism is subject to regulatory oversight and risk assessment by the respective statutory authorities if the recipient organism or the source organism of the introduced genetic elements or traits is known to display risky and/or unwanted characteristics (e.g., plant pathogenicity, weediness, toxicity or pesticidal effects or compositional differences that are not generally recognized as safe).
- The product-oriented regulatory trigger used in Canada can also be considered risk-oriented, although in a different way: novelty of the product indicates a lack of an existing history of safe use, whereas the other trigger component, i.e., the general plausibility of risks related to protection goals as addressed by the biosafety framework, indicates that relevant unwanted risks may exist, which cannot be discounted without a scientific assessment.
- Frameworks which implement process-oriented triggers refer in an indirect way to risks associated with the products established by genetic modification techniques. As stated in Directive 2001/18/EC only products created with techniques, which “*have conventionally been used in a number of applications and have a long safety record*” should be exempt from the Directive. In line with a precautionary approach the wider range of genetic modifications which may be introduced by GM-technology, the limitations of existing knowledge concerning the potential effects of a GMO and the difficulties to address the complex interactions of modified organisms with the environment are important reasons for conducting a risk assessment prior to use.

In all analyzed regulatory frameworks decisions to determine the regulatory status of particular products are typically based on legal and/or technical interpretations of the definitions of regulated products and the scope of exemptions included in the respective legislation. An evaluation of the specific hazards associated with a particular application is not conducted at this stage, but only during the risk assessment of applications which are subject to a specific biosafety framework.

### Implementation and Interpretation of Different Trigger Definitions Results in Heterogenous Regulatory Scopes

Relevant differences can be seen in both classes of triggers (process- and product-oriented). As a result different ranges of organisms and products are being regulated by the different national biosafety frameworks.

The product-oriented regulatory triggers in the USA and Canada biosafety legislation differ significantly from each other:

- The US trigger for the regulation of environmental release applications relates to specific risk issues as outlined in the Coordinated Framework for the Regulation of Biotechnology, e.g., plant pathogenicity, the risks of creating a modified variety of a noxious weed, environmental toxicity of plant protectants (EOP, 2017). The intended focus is on different product-related risks. However, this is not achieved with full consistency in practice. Kuzma (2016b) notes that for the majority of products regulated by USDA-APHIS the process of the employed GM technology, i.e. *Agrobacterium*-mediated transformation, triggers regulation. This results in de facto process-based decisions (Wolt, 2017). The US system is described as “a strange patchwork of rules and exceptions” (Strauss and Sax, 2016) and considered to be a hybrid of process- and product-oriented reasoning (McHughen, 2016; Strauss and Sax, 2016).
- The regulatory trigger implemented by Canada is based on novelty in combination with a given plausibility that these products may have adverse (environmental) effects (Shearer, 2014). The technology used for the generation of a modified organism is therefore irrelevant for the determination of the regulatory status. Since the introduction of the biosafety framework all GM plants have been considered to contain novel traits and have been assessed for environmental safety in Canada (NAS 2016). According to the expert interview this was still the case in late 2017, however this policy could change in the future based on the outcome of an ongoing national review of implementation of the biosafety framework. The scope of the Canadian regulations also covers novel plants derived by non-GM breeding methods like classical mutagenesis. In the USA such plants are only regulated if a trait is associated with one of the specific risk factors mentioned above. In all other analyzed countries plants derived by conventional breeding methods are not subject to the respective biosafety laws.

Process-oriented regulatory triggers are based on country-specific definitions. Differences exist concerning the specific references made to certain techniques in the definitions of regulated products as well as in the exemptions according to the respective biosafety laws and regulations. The scope and specificity of such exemptions decisively influence the overall range of regulated products. Exemptions can be defined quite specifically as e.g., in New Zealand or more general, e.g., as in the EU biosafety framework. The uncertainty concerning the scope of the exemption of mutagenesis according to the EU Directive 2001/18/EC was only by a ruling of the European Court of Justice (ECJ), which determined that products developed by mutagenesis induced by genome editing are covered by the EU trigger definition. Only products of mutagenesis induced by chemical mutagens or ionizing radiation which have a long safety record are exempt from regulatory oversight according to the ECJ decision (ECJ, 2018). In some countries, the presence or absence of a foreign DNA sequence in the final product is a crucial characteristic which determines if the products are subject to regulation or not. For example in Argentina biotechnology applications which retain no transgenic modifications in the final product (“null segregants”) are not regulated. This is an indication that some process-oriented triggers include features of product-orientation. In summary both systems using either product- or process-triggers show features of the respective other system. This explains the difficulties to unequivocally classify some regulatory frameworks as either product- or process-oriented. As a result the classifications of existing regulatory frameworks according to different authors vary, e.g., as exemplified by the diverging classifications provided by Ishii and Araki (2017) as compared with other analyses, including the study at hands.

### Risk Assessment Is Trigger-Independent, but Takes Into Account the Process of Development

The risk assessments conducted under all legislations address similar general goals, i.e., to identify adverse effects on human and animal health as well as on the environment. All frameworks require that a case-specific problem formulation is conducted to identify specific risk hypotheses for the individual products/organisms. The problem formulation needs to address relevant risk issues, associated with the characteristics of the regulated products or organisms, i.e., the new trait(s), the modified organism as a whole, and its interaction with the receiving environment. None of the analyzed regulatory frameworks, including frameworks with process-oriented triggers, is specifically focusing the risk assessment on technology-related issues. However, all frameworks, including the ones with product-oriented triggers, consider technology-related issues in the course of the risk assessment process. Most frameworks, including the ones in Canada and the USA, require specific information on methods applied during development, usually in the context of molecular characterization of the assessed products. Canadian authorities also initiated and conducted research projects aimed at elucidating and characterizing method-related unintended effects relevant for risk assessment (Ladics et al., 2015).

Thus, we cannot make a clear distinction between systems with either product-oriented or process-oriented regulatory triggers regarding their general approaches to risk assessment. Therefore, we argue that the terms “process-oriented” and “product-oriented” only apply to the type of regulatory triggers. In our opinion these terms should not be used otherwise, e.g., in relation to approaches used for risk assessment as implied in some previous publications (Schuttelaar, 2015; McHughen, 2016; Ricroch et al., 2016), since risk assessment in the different biosafety frameworks is conducted independent of the particular nature of the regulatory trigger (Kuzma, 2016b).

While the general principles and approaches to risk assessment applied in the analyzed countries are comparable and independent of the implemented regulatory triggers, the specific (data) requirements and the extent of the risk assessment requirements vary between legislations. e.g., not all biosafety frameworks mandate a comprehensive assessment of indirect and long-term effects similar to the approach implemented in the EU. Likewise additional regulatory requirements, which are implemented in correspondence to the results of risk-assessment, like risk management (e.g., conditions for use to address identified risks) and monitoring (including general surveillance for unanticipated effects) are applied to different degrees in the countries analyzed in this study (see Table 1). For countries which decided to implement a comprehensive set of requirements for applications subject to the respective biosafety frameworks it does therefore matter very much if a particular application is found to be covered by the biosafety framework or not. Therefore, the extent of consistency regarding the level of scrutiny which is provided for individual applications is closely tied to the particular details of the regulatory trigger which is applied in a given country.

## Approaches to Regulate nGM Applications

The countries analyzed in this study have different levels of regulatory experience concerning applications developed with nGMs (Table 2). In all countries nGM approaches and particularly genome editing are used in basic and applied research. However, most nGM applications are still being developed in confined facilities. In a number of countries, including the EU, field testing of different nGM applications (e.g., products developed by genome editing, cisgenesis and null-segregant technology) is under way.

**Table 2:** Regulatory aspects related to nGM applications.

**Country**	**Current regulatory approach**	**Policy development regarding nGMs**	**Focus of policy amendments**	**Current experiences with nGM applications**
European Union	Determination if specific types of nGMs are subject to GMO legislation	No amendment of Directive 2001/18/EC proposed by Europ. Commission, but Europ. Court of Justice ruled that directed mutagenesis is subject to GMO legislation (ECJ 2018)	–	No experience on European level with applications for unconfined release and placing on the market; however field trials with some nGM applications are conducted (SAM, 2017)
Argentina	Determination if nGM product is subject to GMO legislation	Supplementary resolution adopted 2015 providing criteria for case-by-case decisions (Resolution No. 173/2015)	–	Until June 2018 12 requests were evaluated according to Resolution No. 173/2015, incl. 10 applications of genome editing, mostly in plants, mostly not regulated (OECD, 2018)
Australia	Determination if nGM process is subject to GMO legislation	OGTR proposed technical amendments to legislation, consultation in progress	Genome editing (SDN-1)	No applications for unconfined release; field trials with some nGM applications are conducted
Brazil	Determination if nGM product is subject to GMO legislation	Supplementary resolution adopted in January 2018 (Normative Resolution No 16) comparable to supplementary regulation in Argentina)	–	Use of nGMs in contained use facilities; two yeast lines modified by genome editing were evaluated according to Resolution No 16 (not regulated)
Canada	Determination if individual nGM product is novel	Review of risk assessment requirements initiated	–	Several applications authorized (e.g., cisgenic potato, genome edited oilseed rape)
New Zealand	GMO legislation is currently applied for all nGMs	Government adopted policy to direct technical ruling by NZ-EPA, no immediate policy changes foreseen	GMO legislation only exempts chemical or radiation induced mutagenesis	Use of nGMs for research and development activities; some genome editing determined to be regulated
Norway	Determination if specific types of nGMs are subject to GMO legislation	Technical discussions to inform further steps (following EU approach)	–	No applications for unconfined release submitted; use of nGMs in contained use facilities
South Africa	GMO legislation is currently applied for all nGMs	Discussion on policy amendment ongoing	–	No applications for unconfined release submitted; use of nGMs in contained use facilities
Switzerland	Determination if specific types of nGMs are subject to GMO legislation	Stakeholder discussions to inform future policy	–	No applications for unconfined release; field trials with some nGM applications are conducted
USA	Determination if individual nGM product is regulated	Consultations on policy to deregulate certain techniques (e.g., cisgenesis)	–	Several decisions to exempt nGM applications from regulation; a number of nGM applications in regulatory review

Technological developments are rapidly expanding the range of available nGMs, e.g., of methods increasing the range of possible traits and the speed of development. This means that the regulatory bodies will be confronted with a growing range of nGM applications and products with different characteristics (OECD, 2018).

At the present time, the available practical experience with the regulation of nGM applications and the determination of the regulatory status of individual nGM applications is still quite limited. For the time being most of the requests to determine the regulatory status of nGM applications were received by the authorities in the USA, Argentina and Canada.

### Determination of the Regulatory Status for nGM Applications

Differences exist between the countries regarding the determination of the regulatory status of an application, (i.e., the initial decision on whether a particular product, e.g., an nGM application, is covered by the respective biosafety legislation or not).

Only a few countries, e.g., Argentina, Brazil, Canada, New Zealand, and the USA, include provisions that lay down specific procedures for the determination of the regulatory status of applications in their biosafety legislation. In other regulatory frameworks, particularly in the EU, a significant level of uncertainty remained about the regulatory status of nGM applications (Jones, 2015b; Sprink et al., 2016b; Wolt et al., 2016a). In the absence of a specific policy to address this uncertainty the developers have to consult the respective competent authority about the status of individual products or request that these authorities determine a status of regulation. Some authorities, e.g., in Australia and in Canada, actively recommend that developers address any unclear issues during pre-submission consultations.

In some EU Member States, e.g., Germany, UK, the Netherlands, and Sweden, developers have approached the authorities with requests to determine the status of different plants developed by genome editing (BVL, 2015; Jansson, 2018). These decisions, e.g., concerning herbicide resistant oilseed rape lines developed by ODM, were based on an interpretation of the GMO definition given in Article 2 of Directive 2001/18/EC which argues that the expression “ … *organism, …, in which the genetic material has been altered in a way that does not occur naturally …”* refers to the characteristics of the genetic modifications in the final product rather than to the methods used for genetic modification (BVL, 2015; Sprink et al., 2016b; Kahrmann et al., 2017). However, these decisions were taken prior to the ECJ ruling on applications of directed mutagenesis published in July 2018. The ECJ determined in its ruling that applications of directed mutagenesis are covered by the regulatory trigger implemented by Directive 2001/18/EC in the EU and also provided the interpretation that they are not exempted according to Article 3, Para 1 and Annex 1B of the directive. The court concluded that the exemption of mutagenesis methods referred to in Annex 1B does not apply to the introduction of genetic modifications by nGMs like genome editing, since the risks linked to the use of those new genetic modification techniques/methods of mutagenesis might prove to be similar to those which result from the production and release of a GMO through transgenesis (ECJ, 2018). The ruling confirmed that a general exemption of new methods for mutagenesis would not be in line with obligations for regulatory oversight and risk assessment in accordance with the precautionary principle enshrined in European legislation. Consequently any previous decisions taken by authorities of EU member states have to be reviewed and repealed when not in line with the ECJ ruling.

According to the different regulatory triggers employed in other legislations, some countries, e.g., the USA, have decided otherwise when dealing with applications developed by genome editing and other nGMs. One of the US authorities, USDA-APHIS, operates a service dedicated to answer inquiries about the regulatory status of specific products according to Title 7 CFR part 340. The application letters and results of the “Am I regulated?”-process are made available to the public via a dedicated website (USDA-APHIS, 2018). More than 30 “Am I regulated?”-inquiries were submitted between 2011 and May 2018 for products developed with different nGMs including genome editing, cisgenesis (or offspring from cisgenic plants), and null segregants (developed for epigenetic engineering, accelerated breeding and chromosome elimination purposes). 16 applications of genome editing were evaluated, mostly SDN-1 applications. 7 of these applications were developed with CRISPR-methods. The inquiries concerned applications for a variety of intended traits (disease-resistance, compositional modification, drought tolerance, salt tolerance, and modified developmental characteristics such as delayed flowering) in major crops (including maize, wheat, soybean, rice, and potato) as well as in plants like tomato, tobacco, alfalfa and wild foxtail millet, apple trees, and plum trees. As also noted by Waltz (2018) most of these applications are not subject to regulation by USDA-APHIS, since no sequences derived from plant pathogens are introduced during their development and the modified plant species are themselves not known to be plant pathogens or noxious weeds. Therefore, no regulatory oversight or risk assessment will be provided by USDA-APHIS for these applications (Waltz, 2018). Only one application (a cisgenic scab-resistant apple) has been found to be subject to regulation by USDA-APHIS so far, due to the fact that *Agrobacterium tumefaciens*, a known plant pathogen, was used as a vector agent for transformation.

However, the inquiries addressed to USDA-APHIS do not necessarily indicate that the above mentioned products can be expected to be commercialized in the near future. Rather they only indicate that the developers are interested in further development of these products, including field testing. Of better predictive value for commercialization in the near future are the statements by the US Food and Drug Administration (FDA) concerning the results of the (voluntary) consultations of developers with FDA concerning the food safety of their products. However, as of July 2018 only a few respective FDA statements have been published, mostly addressing potato lines with increased disease resistance and altered composition (FDA, 2018).

Thus, only limited experience is available so far related to regulatory oversight for nGM applications according to the existing biosafety frameworks and in particular with case-specific risk assessment of such applications.

### Transparency Concerning the Status of Regulation of nGM Products Is a Crucial Issue

Transparency in decision-making is an important issue for all regulatory frameworks which implement process- or product-oriented regulatory triggers. This is acknowledged by regulators from all countries that have been investigated for this study. Most of the biosafety frameworks do not provide the means for ensuring transparency. Only the regulatory decisions taken by USDA-APHIS in the USA in response to the inquiries for the status of regulation are made publicly available irrespective of whether to the products were found to be subject to regulation under Title 7 CFR part 340 or not. In other countries, including Canada, transparency is typically provided only for those applications which are covered by the respective biosafety legislation, e.g., the PNT regulation.

However, informing the public about the regulatory status of biotech applications and in particular of nGM applications is regarded as a matter of crucial importance. Even experts calling for decreasing the level of regulatory oversight of biotechnology applications in the USA support that a registry of all applications should be established and maintained (Strauss and Sax, 2016). Such a registry should also include applications which have differing regulatory status in varies countries (e.g., SDN-1 in Argentina, Brazil and the USA compared to the EU and New Zealand). With a view to international trade and the varying regulatory status of comparable nGM applications, access to this information will be highly important.

### Regulatory Approaches Addressing nGM Applications

The countries investigated in this study including the EU have not implemented specific regulations for nGM applications, which are independent from the existing regulatory biosafety frameworks for GMOs.

Only Argentina and Brazil have passed supplementary legislation (Normative Resolution No. 173/2015 and Normative Resolution No. 6/2018, respectively) to better address regulatory issues associated with nGM applications (Whelan and Lema, 2015; OECD, 2018). These resolutions outline procedures and criteria for the determination of the regulatory status, which can be applied by the competent authority to decision making on submissions of individual nGM applications. Until June 2018 12 requests concerning the regulatory status of different nGM applications mostly for modified plants were evaluated in Argentina according to Resolution No. 173/2015, including 10 applications of genome editing, Most of these applications were found not to be subject to the Argentinian biosafety law.

In the absence of a general policy on nGM applications, other countries which implement process-oriented regulatory triggers are also facing the challenge to make decisions on the regulatory status of individual nGM applications. The determination of the regulatory status may be initiated by a specific inquiry about an individual nGM application addressed to the respective competent authority, as happened for herbicide-resistant oilseed rape produced by ODM in several EU member states like Germany and Sweden. The case also led to the recent ECJ proceedings and the ECJ ruling provided a binding legal interpretation of the current EU legislation which determined that all genome editing applications are subject to Directive 2001/18/EC and thus the EU biosafety framework.

In order to better define the status of nGM applications and to amend or change legislation accordingly, various countries, among them the EU, USA, and Australia, have performed general and nGM-specific policy reviews of existing regulatory approaches or started to conduct such reviews, e.g., as in Australia (LGFGT, 2018). National discussions on nGM applications involving policy makers, regulatory bodies, technical expert groups, scientific academies, and a wide range of other stakeholders and public consultations are also conducted in other countries.

The discussions concerning general or technical amendments of existing legislation are currently at different stages in the countries included in this study. Australia is a good example to illustrate that this process can be associated with some challenges. Different Australian institutions are currently conducting several parallel reviews of different elements of its biosafety framework:

- The Australian Office of the Gene Technology Regulator (OGTR), which oversees the environmental release of GMOs, has proposed technical amendments to the existing definitions of the GMO regulations to better address nGM applications. According to the proposed amendment SDN-1 type genome editing applications, should not be regulated in the same way as GMOs (OGTR, 2018).
- Food Standards Australia and New Zealand (FSANZ), a shared authority between Australia and New Zealand, is currently conducting a parallel consultation process to gather input from scientists, developers and the public concerning the regulation of foods derived using different nGMs (FSANZ, 2018a). The aim is to determine if a concrete proposal should be developed to amend the Food Standards Code and to determine which changes would be reasonable and acceptable for the various stakeholders (FSANZ, 2018b).
- Further general amendments to the overall framework in Australia might be proposed in the course of the ongoing third review of the national gene technology regulatory framework (LGFGT, 2018).

An important issue recognized by FSANZ (2018b) is the importance that any amendments addressing different elements of the regulatory framework should be aligned to achieve coherence of what is regulated as GMO for environmental release purposes and what is regulated as food produced using gene technology in Australia and New Zealand. However, this will not be easy to achieve. First of all a different range of technologies is addressed by OGTR in Australia, by NZ-EPA in New Zealand and by FSANZ at the binational level. Secondly OGTR in Australia and NZ-EPA in New Zealand are likely to implement different regulatory approaches vis a vis applications of various types of genome editing (NZ-EPA regulating all applications of genome editing, while OGTR may only regulate SDN-2, SDN-3, and ODM applications). Therefore, overall consistency between specific regulations for applications for environmental release and for food safety can hardly be achieved.

Furthermore, the issue remains whether the future amendments in Australia will be able to ensure that products with similar characteristics will be subject to similar regulatory requirements. The OGTR states that the proposed technical revision “best supports the effectiveness of the legislative framework”(OGTR, 2018). However, an implementation of such a proposal will not achieve that plants with comparable genetic modifications and traits are consistently addressed by similar risk assessment: according to the proposal all SDN-2 and ODM applications would be subject to risk assessment according to the Australian Gene Technology Act and Regulations, whereas all SDN-1 applications would not. The two product classes would thus be regulated differently even if certain applications from either class would contain similar genetic modifications or traits.

New Zealand introduced a clarification of the law as a response to a court decision on specific applications of genome editing (Kershen, 2015): Only products developed by chemical or radiation induced mutagenesis are exempted from regulation. The New Zealand government has also decided that for the time being all nGM applications are regulated according to the national biosafety framework. In the EU the recent ruling of the ECJ has clarified the current regulatory status of nGMs for genome editing in a similar way (ECJ, 2018).

Taking decisions on individual applications (i.e., case-by-case) is the default for determining the regulatory status in biosafety frameworks implementing product-oriented regulatory triggers, i.e., the USA and Canada.

Overall it can be concluded that all of the analyzed countries are discussing similar questions and that most of them face comparable challenges, which are including but not limited to:

- Ambiguities of the trigger definitions (process and product) (all countries)
- Inconsistent coverage of products associated with comparable risks (EU, other countries with process-oriented regulation, USA)
- Lack of suitable criteria for the determination of the regulatory status (most countries).

Concerning the regulation of applications developed by genome editing (in particular for SDN-1, SDN-2, SDN-3, or ODM-based techniques) different approaches are or may be taken in the future in the countries covered in this study:

- No biosafety oversight of genome editing applications, if no genetic elements from pathogenic species or pesticidal traits are introduced: USA (applications for environmental release).
- Regulation of SDN-3 applications involving recombinant DNA constructs, but not of SDN-1, SDN-2 or ODM applications (that do not contain any recombinant DNA): Argentina, Brazil.
- Regulation of ODM and SDN-2, but not of SDN-1 applications: Australia (according to the proposed revision of the Gene Technology Regulations).
- Coverage of all types of genome editing if they are used to develop plants with novel traits: Canada.
- Coverage of all types of genome editing approaches: EU, New Zealand.

A whole spectrum of different approaches is used in the analyzed countries. At the one end of the spectrum most genome editing applications, i.e., all applications which do not contain genetic elements from pathogenic species or pesticidal traits, are excluded from biosafety oversight in the USA. At the other end of the spectrum, all types of genome editing are covered by the existing biosafety framework, either irrespective of the nature of the traits developed by genome editing (EU, New Zealand), or for all applications containing novel traits (Canada).

Other countries have introduced (Argentina, Brasil) or have proposed to introduce specific criteria (Australia) to determine different types of genome editing applications are or will be covered by the biosafety frameworks of these countries. These criteria are mostly aimed at improving regulatory certainty for authorities and applicants. This is done either by providing further clarifications to the trigger definitions included in the respective biosafety laws, e.g., using the presence or absence of recombinant DNA constructs to clarify if a “novel combination of genetic material” was established (in Argentina) or if “genetic engineering technique(s)” were used (in Brasil) or by introducing a clear way to distinguish between different types of genome editing applications(e.g., SDN-1 applications without the use of nucleic acid sequences supplied as repair template(s) *in trans* and SDN-2, SDN-3 and ODM applications which use such template DNA(s) to direct genetic modifications). However, these criteria are not aimed specifically at distinguishing between applications with a different level of associated risk.

## Regulatory Options for nGM Applications

In summary our analysis indicates that the following approaches are used or may be used when countries wish to provide regulatory oversight for nGM applications:

1. Existing regulatory framework for GMOs is applied to nGM applications
  (a) For all nGMs (South Africa) or for certain types of nGMs (EU)
  (b) Based on case-by-case decisions on individual nGM applications (USA, Canada)
2. Technical revision of existing regulations (definitions and exemptions) (New Zealand, Australia)
3. Implementation of supplementary legislation supporting the existing framework to clarify aspects related to regulation of nGM applications (Argentina, Brazil)
4. New stand-alone legislation for nGM applications, in addition to existing legislation for GMOs (option, no example as yet)
5. “New” overall framework for all biotechnology applications (option, no example as yet).

So far, most countries have not introduced specific legal instruments for nGM applications and have been using the existing regulatory framework to deal with them. In countries with product-oriented triggers individual applications are evaluated at a technical level to determine whether they are covered by the criteria included in the respective legislation (option 1b). Authorities and courts from countries (and the EU) which implement process-oriented regulatory triggers have to provide legal interpretations of the existing laws to determine the regulatory status of categories or types of nGM applications (option 1a).

A few countries have introduced amendments to the existing regulatory framework, either technical revisions of existing definitions of regulated products and exemptions included in the current legislation (option 2) or supplementary regulations to introduce procedures and criteria for the determination of the regulatory status of nGM applications (option 3).

Options 4 and 5 have not been used in practice yet. A general new biosafety framework for all biotechnological applications (also including nGM applications) is discussed in Switzerland, however no proposal has as yet been developed.

### Advantages and Disadvantages of Process-Oriented or Product-Oriented Regulatory Triggers for the Regulation of nGM Applications

Are regulatory systems based on either product- or process-oriented regulatory triggers more advantageous for the regulation of nGM applications (Sprink et al., 2016a)? We analyzed the available information and interviewed regulatory experts concerning their views. A non-exhaustive overview on the perceived general advantages and disadvantages of both systems is presented in Table 3.

**Table 3:** General analysis of the advantages and disadvantages associated with product- and process-oriented regulatory triggers as well as the associated challenges concerning implementation of such systems [as particularly relevant for nGM applications (nGMs) or GM applications (GMO)].

**Advantages**	**Disadvantages**	**Challenges**
**PRODUCT-ORIENTED REGULATORY TRIGGERS**		
High flexibility to accommodate products of emerging technologies without need for legislation change (nGMs)	Some product-oriented triggers may result in inconsistent coverage of products with comparable traits (USA: nGMs and GMOs)	Different competent authorities may be involved, if a broad scope of use is intended (env. release and food/feed use)—split responsibilities, need for coordination
Existing regulatory structures can be used for comparable products	Individual applications may need to be reviewed for regulatory status	Criteria and guidance required for decision making on regulatory status
Similar regulatory approach for comparable products developed by different techniques	Process to determine regulatory status considered more complicated and less predictable compared with process-related triggers (GMOs)	Limited compatibility with regulatory systems based on process-oriented triggers regarding the scope of regulated articles
Consistent risk assessment perspective for products irrespective of the method of production	The typical remit of existing authorities may be ill-suited to address risk assessment challenges of emerging applications	
**PROCESS-ORIENTED REGULATORY TRIGGERS**		
Typically new sectoral legislation is introduced and implemented by a specific authority	Limited flexibility to accommodate products of emerging technologies—possible need for legislation change in reaction to technological developments (nGMs)	Severe challenges of trigger interpretation regarding some nGMs if specific guidance is not available
Newly introduced sectoral regulations address all relevant risk assessment requirements	Regulation gaps until newly emerging technologies are addressed by trigger amendments (nGMs)	Ambiguous trigger definitions may lead to interpretation conflicts that have to be settled by administrative and/or court proceedings (nGMs in particular)
Process-oriented triggers considered easier to implement and more predictable (GMOs)	Trigger specifics (exemptions) may result in inconsistent coverage of products with comparable risk (nGMs)	Limited compatibility with regulatory systems based on product-oriented triggers regarding the scope of regulated articles

The analysis shows that both trigger systems have a number of generic advantages and disadvantages. Experience in the analyzed countries demonstrates how important the specific details of implementation of the basic concepts are for the workability of both regulatory approaches. Thus, as noted by Kuzma (2016b) neither system can be regarded as superior at a general level.

However, systems based on product-oriented triggers are considered more flexible when it comes to products developed with newly emerging technologies, without the need to repeatedly adapt existing legislation. Frameworks based on product-oriented triggers may strengthen consistency in the regulation of products with comparable characteristics. This however depends on whether a particular system indeed achieves consistent coverage of products associated with comparable possible risks. The US regulatory framework shows that specific product-oriented trigger definitions can result in an inconsistent range of regulated products: e.g., *Agrobacterium*-mediated transformation results in regulation by USDA, while transformation with similar transgenic constructs of non-plant pathogenic origin by particle bombardment does not (NAS, 2016). The current distribution of responsibilities in the USA between existing authorities also results in emerging biotech products being regulated by authorities that have an inadequate regulatory focus for such products, resulting in particular challenges in addressing issues of greatest concern during risk assessment (Kuzma, 2016a). Product-oriented triggers require a separate determination of the regulatory status for each specific application, which is considered by the interviewed regulatory experts to be more laborious and complex for involved authorities.

A main advantage of frameworks based on process-oriented regulatory triggers is that they provide a clear and straightforward means to establish the regulatory status of classic GMOs both for developers and authorities. The establishment of specific authorities with a consolidated responsibility for all matters of sectoral biosafety legislation can provide a better framework to prevent regulatory gaps and to ensure that a comprehensive risk assessment approach is implemented. These systems however are significantly challenged by several types of nGM applications, particularly products developed by genome editing, if existing definitions are ambiguous. Without concrete policy and appropriate criteria for interpretation, lengthy legal disputes e.g., as in New Zealand and the EU can occur, delaying decisions on individual applications as well as policy development.

An adaptation of process-oriented triggers to ongoing technical developments typically requires the repeated introduction of specific amendments in response to technological developments. Such amendments may need considerable time for their introduction, e.g., for consultation and implementation, and this might cause a temporal regulatory gap for the respective nGM applications. Trigger definitions covering a very broad scope of applications might potentially be flexible enough to avoid the development of regulation gaps, however at the expense of a higher number of applications which need to be assessed for risks by the competent authorities.

Our analysis indicates that the specific details of a particular trigger are more important than the general choice of either a product-oriented or a process-oriented system. The respective differences of implementation result in

- significantly different ranges of regulated products, particularly of nGM products,
- different levels of regulatory uncertainties to determine the status of regulation of specific (nGM) products,
- different levels of consistency to address comparable risks of products developed by different technologies (including GM technology, nGMs and conventional breeding).

Further discussions should therefore not only focus on the question whether a system is based on a process- or product-oriented trigger. The implications of the specific details of existing or proposed trigger definitions on the range of regulated articles also should be taken into account when judging the advantages or disadvantages of a particular system.

It is noted that only some product-oriented systems, like the Canadian Plant with Novel Traits-regulations, implement a similar regulatory approach for all novel products irrespective of the methods used for their development and consistently regulate novel biotech crops as wells as novel plants produced by conventional breeding methods.

### Would Sectoral Regulation Outside the Biosafety Frameworks be Sufficient for nGM Applications to Ensure a Suitable Risk Assessment?

For nGM applications which are subject to any of the biosafety frameworks, the same regulatory requirements, e.g., regarding risk assessment or other obligations, apply as for any other regulated products. However, the USA and Canada currently consider specifying different risk assessment requirements for applications belonging to different risk classes.

Generally biotech products which do not to fall under the provisions of the respective biosafety frameworks are still subject to other regulations addressing agricultural products (e.g., seed and plant propagating materials, animal and plant health, food and feed safety, nature conservation). Our analysis indicates that the general requirements according to such legislation in the different countries are broadly comparable. The following examples of such requirements apply to products of genome editing or other nGM applications in case it is found that these products are not subject to existing biosafety legislation:

- Variety registration regimes are implemented in all countries included in this study as well as globally to ensure seed quality, the distinctiveness and stability of traits, as well as the uniformity of seed lots and a number of safety parameters for certain plant species. These issues are assessed according to international standards (UPOV, 2002).
- The general provisions of food and feed safety legislation in the different countries are also applicable to biotech products. In some countries specific products may also be covered by legislation addressing novel foods, such as Regulation (EU) 2015/2283 in the European Union.
- All of the investigated countries implement phytosanitary measures according to the WTO Sanitary and Phytosanitary (SPS)-Agreement. According to this agreement requirements for pest risk assessment can be implemented based on standards developed by the International Plant Protection Convention (IPPC), as well as weed risk assessments which may be required for newly imported plant propagating material.
- Some countries, in particular Australia and New Zealand, implement quarantine and assessment requirements for organisms which are newly introduced into the respective countries.

A recently published legal opinion analyzed whether existing EU legislation e.g., for seeds, food and feed, pesticides and nature conservation, would provide a suitable framework for the assessment of nGM applications outside the biotechnology legislation for risks to human and animal health and to the environment: Spranger (2017) concluded that such sectoral legislation will not provide a suitable framework for an assessment of nGM applications. A premarket assessment of products is either not generally required (e.g., according to the food law) or the required assessments are unsuitable for replacing the comprehensive risk assessment required by the EU biosafety framework (e.g., for novel food laws, pesticide regulations). This conclusion is supported by the results of another recent study conducted by Voigt and Klima (2017).

Furthermore, some general requirements according to regulations for quarantine, phytosanitary measures and invasive alien species only apply to organisms or species, which are newly introduced into a country.

The information gathered from regulatory experts from non-EU countries indicates that the general conclusion drawn by Spranger (2017) for the EU also applies to all other regulatory systems: The general requirements applicable to the agricultural use of plants in the different countries do not ensure a risk assessment comparable to that according to the respective national biosafety frameworks. This outcome is independent of the type of regulatory trigger implemented in a respective framework and can also affect systems with particular product-oriented triggers like the USA (Kuzma, 2016b; Zetterberg and Edvardsson Björnberg, 2017).

## Conclusions

Our analysis investigated how regulatory systems determine the regulatory status of biotechnology applications. In general two categories of regulatory triggers can be distinguished: process-oriented and product-oriented. The overarching question was which trigger would generally be better suited to address new developments in the field of biotechnology, including different nGMs.

Our review of available scientific literature and the results of the interviews conducted with regulatory experts allows us to draw the general conclusion that in practice neither trigger system can be generally regarded as superior when addressing the challenges posed by nGMs. We note that all existing triggers have generic advantages and disadvantages and that the specific trigger definitions and their implementation are more important when defining the range of covered products than an initial choice of a either a process- or a product-oriented trigger system. On the one hand none of the existing trigger systems allows for a straightforward, unambiguous denomination of regulated articles. In process-triggered systems administrative, legislative or court decisions (like in the EU or New Zealand) are necessary to clarify which categories of nGM applications fall under the respective legislation/GMO definition. In frameworks based on product-oriented triggers nGM applications are scrutinized individually to assign their regulatory status.

On the other hand most of the existing biosafety frameworks do not address newly developed products in a fully consistent manner. What all biosafety frameworks have in common is that they aim to identify and assess environmental and health risks associated with a given product generated by biotechnology. Ideally those frameworks should aim to regulate products with comparable risks in a similar manner. In practice many examples can be identified where products with comparable characteristics are subject to very different requirements. In frameworks with process-oriented triggers products generated with GM-technology need to be assessed for biosafety, whereas comparable products developed with conventional approaches are not required to undergo a similar premarket risk assessment. In the product-oriented framework operated in the USA similar products developed with different transformation methods are treated differently irrespective of similar characteristics of the final product. A higher degree of consistency is currently only achieved in the Canadian framework, which is based on a product-oriented trigger focusing on the novelty of products.

With the advent of nGMs coherent regulation of novel biotechnology products becomes even more challenging. The information gathered in our study indicates that sectoral regulation which applies for all agricultural- and food-products does not provide for a comparable breadth and standard of risk assessment as compared with the requirements according to the respective biosafety frameworks. The decision as to whether certain nGM applications should fall under the respective biosafety frameworks is therefore critical for the scope and the quality of risk assessment which is provided for these applications. This decision is ultimately a political one. With that in mind legislators have different options to regulate nGMs for biosafety purposes, if desired. Those options range from applying and/or adapting existing rules to developing a new overall framework for all biotechnology applications or additional biosafety regulations for nGM applications. The latter would amount to substantial changes of the existing frameworks, specifically for frameworks based on process-oriented triggers. According to the information collated in our study such major legislative changes are not likely to be implemented in any of the investigated countries.

The regulatory status of nGM applications is in the process of being resolved in a growing number of countries by administrative or judicial decisions based on the existing biosafety laws and by introducing supplementary regulations specifying concrete criteria for such decisions. However, the lack of harmonization at the global level concerning such approaches will lead to situations that identical biotechnological applications/products are assigned opposing different regulatory status in different jurisdictions. This will result in a serious challenge for international trade between such countries. To address this challenge transparency in decision-making for nGM applications is a crucial issue acknowledged by regulatory experts from all investigated frameworks. We consider a public international registry which includes all biotech products that are placed on the market, among them (nGM) applications exempted in certain countries from regulatory oversight and risk assessment prior to commercial use, to be essential. This would ensure that all countries are enabled to identify products developed by nGMs, if their respective legislation requires them to do so. Non-registered and undescribed products developed by certain nGMs, e.g., SDN-1 type genome editing, can be difficult to detect and keep track of. Shipment of agricultural products suspected to be of uncertain composition, i.e., containing nGM products, could provoke unwanted disruptions of international trade.

We note that the Biosafety Clearing House (BCH) according to the CPB is an existing registry for GMO applications at the international level that also contains information voluntarily submitted by non-parties to the Protocol. It may also provide an appropriate framework for the purpose of sharing relevant information on nGM applications. We are, however, aware of the fact that it will be a challenge to establish and maintain a registry including nGM applications, which are not subject to regulation according to some national biosafety frameworks, since active voluntary cooperation of country administrations and developers is required. Nevertheless stakeholders from all countries should be aware that sharing information on nGM products will be vital, since global harmonization of regulatory approaches toward applications of genome editing and other nGMs will not be easily achieved in the near future.

## Author Contributions

MFE conducted the study and drafted the manuscript. ME, AH, SS, and HT contributed to the study design and edited the manuscript. All authors have read and approved the manuscript for publication.

### Conflict of Interest Statement

The authors declare that the research was conducted in the absence of any commercial or financial relationships that could be construed as a potential conflict of interest.

## Footnotes

^1^Department of Health and Human Services, Food and Drug Administration [Docket No. FDA-2016-D-4482]. *Clarification of the Food and Drug Administration and Environmental Protection Agency Jurisdiction Over Mosquito-Related Products; Guidance for Industry*. Federal Register Vol. 82. Available online at: https://www.govinfo.gov/content/pkg/FR-2017-10-05/pdf/2017-21494.pdf
