# Supplementary material for: Plants Developed by New Genetic Modification Techniques—Comparison of Existing Regulatory Frameworks in the EU and Non-EU Countries
Source: Front Bioeng Biotechnol. 2019 Feb 19;7:26. doi: 10.3389/fbioe.2019.00026 (PMC6389621; doi:10.3389/fbioe.2019.00026)
Supplement: Supplementary file 1 [file Data_Sheet_1.PDF]

Plants developed by new genetic modification techniques - Comparison of existing regulatory frameworks in the EU and non-EU countries and the respective requirements for risk assessment

Michael F. Eckerstorfer*, Margret Engelhard, Andreas Heissenberger, Samson Simon, Hanka Teichmann

Supplementary information on interviews with regulatory experts

To supplement the available published information qualitative interviews were conducted with regulatory experts from non-EU countries included in this study. The selected experts are involved in the respective national biosafety regulation in different capacities, e.g. as regulators, members of institutions or committees involved in risk assessment of GM applications or stakeholders with a high familiarity with the respective legislation and its implementation and being involved in the process of risk assessment of regulated products (Annex 1). The interview partners answered the questions in their personal capacity based on the understanding that no transcripts of the interviews will be published nor direct quotes from the interviews will be referenced to specific persons. Notes taken during interviews were exchanged with the interview partners for back-checking to avoid misunderstandings. The final information was used as background information to check previously published information for correctness and validity (as of September 2017).

In a few cases appropriate interview partners could not participate due to various reasons (e.g. availability, no national clearance to discuss ongoing internal policy review processes publicly). Alternative interview partners or sources of information are indicated in Annex 1.

A common questionnaire was used to conduct the interviews. The questions were aimed to establish a better understanding of the different regulatory approaches and to learn from experiences gathered in other countries with the practical implementation of the respective regulations. The used questionnaire is included in Annex 2 below. Depending on the current situation in the respective countries, not all questions were equally relevant for all countries and/or could not be addressed by the interview partners.

The term “new genetic modification techniques” (nGM) is used in the following, synonymous with “new techniques” or “new plant breeding techniques” (in reference to nGM plants) as used in other sources.

The questions addressed two general issues:

a) Part 1: Aspects of the existing system developed for GMO regulation in a respective country, in particular:

- How is the regulation "triggered", i.e. which characteristics of the resulting products or biotechnology techniques used are relevant to determine whether a certain application is regulated or not (e.g. is a process-oriented or product-oriented trigger used)?
- Which risk assessment requirements according to the existing biosafety regulatory framework apply for regulated items?

b) Part 2: Issues related to the regulatory treatment of emerging biotechnology-/nGM-applications:

- Which approach is developed or implemented towards nGM-applications?
- Which risk assessment requirements will be applied for nGM-applications?

Annex 1: List of Interview-Partners

	Institution(s) approached	Full interview	Other means of information
Argentina	Ministerio de Agroindustria, Biotechnology Directorate	No<br>(no response)	Information presented to OECD WG on Harmonisation of Oversight in Biotechnology and Conference June 2018 (OECD 2018)
Australia	Office of the Gene Technology Regulator	Yes	-
Brazil	National Technical Commission on Biosafety (CTNBio)	Yes	-
Canada	Canadian Food Inspection Agency, Plant Biosafety Office	Yes	-
New Zealand	NZL-EPA, New Organisms Unit<br>Sustainability Council NZL	No (no availability)<br>Yes	-
Norway	Ministry of Climate and Environment<br>Miljødirektoratet (DIRNAT)	Yes	-
South Africa	National Department of Science and Technology (DST), Directorate of Biotechnology	Yes	-
Switzerland	Federal Office for the Environment (FOEN)	Yes	-
USA	USDA-APHIS, Biotechnology Regulatory Services<br>US-EPA	No<br>(no government clearance)	Information presented to OECD WG on Harmonisation of Oversight in Biotechnology and at Conference June 2018 (OECD 2018)

Annex 2: Questionnaire used for interviews

Opening question:

- How are you involved in the regulation and risk assessment of biotech products in your country?

Part 1

Trigger for regulation of biotech applications:

- Who is making decisions whether specific biotech application are subjected to existing regulations in regulatory practice?

- Which considerations are used to establish the regulatory status of applications?
  (in other words: What triggers regulatory oversight?)
  (trigger related to i) process of generation, ii) product-characteristics, iii) specific risk considerations or iv) a combination of different triggers? Novelty?)

- Are risk-issues considered to establish the regulatory status? (How?)

- Question for systems with product-oriented triggers)

Can you give examples, which applications are regulated or not?
(e.g. are conventional herbicide resistant plants regulated in your country and if so on what basis?
(Which applications are additionally regulated/not regulated in comparison with the scope of regulation in EU/Cartagena Protocol?)

- Are decisions concerning regulation of biotech applications made public? (How?)

Regulatory proceedings:

- What is the remit of competent authorities for regulated products/organisms?
  (If more than one authority is involved: Cooperation between involved authorities?)
  How are the opinions of different involved authorities taken into account in overall decision making? May opinions be disregarded? Does that happen in practice?)

- Which procedure is implemented? (What are main steps in the regulatory pathway?)

- Which specific requirements apply to regulated applications?
  (notification time-limited) authorization/ – (renewal, risk assessment, risk management, monitoring*)*
- What goes well and what is difficult? Which requirements are easy or difficult to implement?

Regulatory environment:

- Which other general regulations apply to biotech applications?
  (What are the objectives of these regulations?)

- Who is responsible for implementation?
  (Cooperation between involved authorities?)
  What are the basic principles for regulation? (precautionary approach, liability law?)

- Is a further development of the regulatory framework in your country discussed currently?
  (Which issues/developments triggered these discussions and what is their focus?)

Part 2

Regulation of nGMs / Scope of regulation

- Which nGM applications are regulated and how are the mentioned examples of nGM applications regulated? (cf. above list of techniques)
  Are decisions based on general administrative provisions in existing legislation?

- Are specific regulations or existing regulations applied to regulate nGMs?
  (GMO regulations, other regulations? – e.g. for conventional breeding products)

- Are decisions based on the definition of GMOs in specific regulations?
  (Was this definition amended with a view to nGMs?)

- How is the definition of regulated items (GMOs) interpreted in practice?
  (e.g. as regards introduction of foreign DNA, new combinations of genetic information, intermediary transgenic modifications, somatic transgenic modifications (agroinfiltration, grafting),

- Will existing regulations cover application of emerging technologies or is there a specific range of covered applications?
  (Which? Examples - Genome editing, e.g. GE without transgenic modification?
  Serial/multiplexed applications?)

Risk assessment (RA) approach:

- Is a risk assessment conducted for nGMs?
  (Is it a (mandatory) requirement for authorisation?)

- Which entity is subject to RA ("event", variety)

- Are any applications exempted from RA? (Which?)

- How is the RA conducted?
  Is a tiered system implemented – e.g. preliminary RA, comprehensive assessment (of specific issues)
  Do specific standards apply for some applications (different information elements, different information requirements)?

- Which issues are considered during RA?
  (technology-oriented considerations?, product-specific considerations? – How is "product"

defined as regards RA? - GMO event/ variety)

- Are benefits considered during RA? (or otherwise, e.g. for decision-making?)

- Is there /Will there be/ an environmental monitoring for nGM applications and how is it designed?

Challenges for implementation:

- Which (specific) challenges are associated with the implementation of national regulations?

- What is done to address these challenges?
  What could be done to support implementation? (in principle? / considering practice?)

- Is the ability to identify nGM products an issue?
  What approaches are used for identification?

- Are there efforts to establish new (specific) regulations or amend existing regulations? (Which ones? State of proceedings? Timeframe for coming into force?)

- Are international harmonisation issues discussed in your country with respect to nGMs? As for example WTO compliance?

- How do you deal with imports from countries that do not regulate nGM applications?
